# Comparing the cost-effectiveness of MRSA control strategies between ICU and non-ICU settings

**DOI:** 10.1186/1753-6561-5-S6-O74

**Published:** 2011-06-29

**Authors:** J Robotham, N Graves, B Cookson, A Barnett, J Wilson, J Edgeworth, C Worby, B Cooper

**Affiliations:** 1 HPA, London, UK; 2 QUT, Brisbane, Australia; 3 Imperial NHS Trust, London, UK; 4 Guy's & St Thomas NHS Trust, London, UK; 5 MORU, Bangkok, Thailand

## Introduction / objectives

Many strategies are used to control MRSA in hospitals. Only a few have been assessed in clinical trials and it is not obvious how findings should be generalised between settings. Uncertainty remains about which strategies represent the most appropriate use of scarce resources. We assess the cost-effectiveness of alternative MRSA screening and infection control strategies in England and Wales and discuss international relevance.

## Methods

Models of MRSA transmission in ICUs and general medical (GM) wards were developed and used to evaluate different screening methods combined with decolonisation or isolation. Strategies were compared in terms of costs and health benefits (quality adjusted life years, QALYs). Different prevalences, proportions of high risk patients and ward sizes were investigated, and probabilistic sensitivity analyses (PSA) conducted.

## Results

Decolonisation strategies were cost-saving in ICUs at a 5% admission prevalence, with admission and weekly PCR screening the most cost-effective (£3,929/QALY). In ICUs, screening and isolation reduced infection rates by ~10%. With admission prevalence ≤5%, targeting screening and isolation to high risk patients was optimal. In GM wards decolonisation and isolation strategies, though able to reduce MRSA infection rates up to ~50%, were not cost-effective.

## Conclusion

The largest reductions in MRSA infection were achieved by screening and decolonisation strategies, and were cost-effective in ICU settings. In comparison, there is limited potential for screening and control strategies to be cost-effective in GM wards due to lower infection and mortality rates.

## Disclosure of interest

None declared.

